# Gastrointestinal Bleeding in Children: The Role of Endoscopy and the Sheffield Scoring System in a Resource-Limited Setting

**DOI:** 10.1097/PG9.0000000000000369

**Published:** 2023-10-05

**Authors:** Oluwafunmilayo Funke Adeniyi, Olufunmilayo Adenike Lesi, Emuobor Aghoghor Odeghe, Ganiyat Oyeleke, Nicholas Croft

**Affiliations:** From the * Department of Paediatrics, College of Medicine, University of Lagos/Lagos University Teaching Hospital, Lagos, Nigeria; † Department of Medicine, College of Medicine, University of Lagos/Lagos University Teaching Hospital, Lagos, Nigeria; ‡ Barts and the London School of Medicine and Dentistry/Queen Mary University of London, United Kingdom.

**Keywords:** pediatric, hematemesis, hematochezia, oesophagogastroduodenoscopy, colonoscopy, stratification system

## Abstract

**Objective:**

To document the clinical presentation, endoscopic diagnosis, and Sheffield scores of children with gastrointestinal (GI) bleeding who were referred for endoscopy at the Lagos University Teaching Hospital. The participants who needed endoscopy based on clinical criteria and according to the Sheffield scores were also documented.

**Methods:**

This study analyzed the records of 111 children with GI bleeding retrospectively from January 2013 to January 2021, while 9 children were recruited prospectively from February 2021 to March 2022. Receiver operating curves and area under the curve were generated to test the ability of the Sheffield scores to predict rebleeds, mortality, and the need for endoscopic intervention for upper GI bleeds.

**Results:**

One hundred and twenty participants were recruited. Ninety-one (75.8%) presented with upper GI bleeding (UGIB), while 29 (24.2%) had lower GI bleeding (LGIB). Only 70 (58.3%) (53 UGIB and 17 LGIB) had endoscopy performed. For UGIB, 5 (9.4%) had no source of the bleeding identified at endoscopy, 12 (22.6%) had variceal bleeding, and 36 (67.9%) had nonvariceal bleeding. Colonoscopy revealed juvenile polyps in 5 (29.4%), indeterminate colitis in 5 (29.4%), ulcerative colitis in 4 (23.5%), Crohn’s disease in 1 (5.9%), and hemorrhoids in 2 (11.8%) participants, respectively. The Sheffield score was ≥8 in 42 (46.1%) of the participants who presented only with UGIB (hematemesis and melena). The scores were significantly related to the type of bleeds, rebleeds, and deaths (*P =* 0.00).

**Conclusion:**

The clinical and endoscopic findings in this study are similar to those reported previously. The Sheffield scoring was useful in assessing Nigerian children. However, due to limited access and other restraints, endoscopy was not performed on all the study participants even when the scoring system was suggestive. The availability, and therefore, utility of GI endoscopy in this setting are still suboptimal. The need for the provision of adequate equipment and resources and the training of personnel is thus recommended.

**WHAT IS KNOWN**

- Gastrointestinal (GI) endoscopy and stratification scoring systems have enhanced the management of GI bleeding, even in the pediatric age group.
- The use of these techniques and scoring systems is yet to be extensively documented in children, especially in the developing world.

**What Is New**

- This study provides comprehensive data on clinical and endoscopic findings on GI bleeding in children from a typical low-resource setting.
- The use of Sheffield scoring system can be utilized as a screening tool to stratify children with GI bleeding in this setting and establish the need for endoscopic interventions.

## INTRODUCTION

Pediatric gastrointestinal (GI) endoscopy presently plays a significant role in the management of GI bleeding especially in the developed world (1–6). To stratify patients with the disease, scoring systems have also been devised. These scoring methods enable stratification in terms of the severity of the bleed, the need for endoscopic evaluation and intervention, the risk of rebleeding, and mortality in such patients (6–9). In the adult population, scoring systems that have been found to be useful include the Rockall and Glasgow-Blatchford systems (6–8). However, these systems have limited clinical utility in the pediatric age group as the etiology and presentation of GI bleeding differ in pediatrics and adults (10–13). The Sheffield scoring system has been found to be particularly useful in children (14) and has been observed to have high specificity (90%), sensitivity (90%), and positive predictive values (81%) (14). However, there is a rarity of studies on the use of this system in African children, especially in the West African sub-region; it has also only been validated for upper GI bleeding.

In terms of endoscopic findings in GI bleeding, there is also a paucity of data in Nigerian children and adolescents, where there is limited access to endoscopic procedures. Additionally, most of the available studies are on adults, with findings in upper GI bleeding (UGIB) rather than in lower GI bleeding (LGIB) (15–17).

This study aims to document the clinical presentation and endoscopic findings in children with GI bleeding referred for endoscopy at Lagos University Teaching Hospital (LUTH). The utility of the Sheffield scoring system in these children for predicting outcome was also documented in those with upper GI bleeds, and as a pilot, we attempted to report a version of the Sheffield score (with adaption below) in lower GI bleeds to see, if possible, to be clinically useful.

## METHODS

This study was conducted at the endoscopy unit of LUTH a multi-specialist tertiary government hospital in Lagos, south-west Nigeria.

### Study Design and Study Population

This study was a descriptive cohort study (both retrospective and prospective in nature), including all children ≤18 years with GI bleeding who were referred for possible endoscopy from January 2013 to March 2022.

The retrospective data were a review of the clinical and endoscopic records from January 2013 to January 2021, and the prospective cases were recruited from February 2021 to March 2022. Children with incomplete data were excluded from the study.

### Data Collection

The details of each study participant were initially entered into a standardized proforma and subsequently transferred into an SPSS version 25(18) spreadsheet. Data obtained included socio-demographic (age, gender, and socioeconomic status), clinical, and endoscopic data. The socioeconomic status of the subjects was categorized using the Ogunlesi socioeconomic classification. (Supplemental Digital Content Appendix1, http://links.lww.com/PG9/A136)

### Clinical Data

Data retrieved from the clinical records included the type and duration of bleed, comorbid conditions, medications used before presentation, vital signs, including capillary refill time, relevant clinical features, and the presence of shock. Hemoglobin/packed cell volume on admission was recorded, and Helicobacter pylori (H.pylori) status if done was also noted. The final outcome for each participant was categorized into: survived, rebleeds/readmissions, or death. Similar clinical data were also documented for those recruited prospectively at the time of presentation. The Sheffield scores for the participants recruited prospectively were also determined and entered into the proforma before the endoscopic procedure.

### Determination of Sheffield Scores

The Sheffield score was recorded for each of the study participants with UGIB, and the score was calculated using the parameters obtained from the history, clinical assessment, laboratory findings, and management/resuscitation (14). (Supplemental Digital Content Appendix 2, http://links.lww.com/PG9/A136).

In the Sheffield score of 24 points, 2 of the criteria (1 point each) are specific to upper GI bleeds, and the majority of the score (22/24) applies to both upper and lower GI bleeds. To allow exploratory examination of the Sheffield score for lower gastrointestinal bleeding (LGIB) we elected to assume the parameter labeled as ‘massive hematemesis’ was the equivalent of massive red blood loss per rectum.

A maximum score of 24 is attainable and the cutoff ≥8 was regarded as a significant score indicating severe bleeding requiring endoscopic evaluation and intervention.

The study participants were stratified according to the type of bleed and age groups using the Sheffield score into the 2 subcategories, that is, those with scores <8 and those with scores ≥8. The relationship between the Sheffield scores, type of bleeds, rebleeds, mortality, and endoscopic procedure was also documented.

### Endoscopic Data and Procedure

Details of the endoscopic procedure were retrieved from the register of the endoscopy unit/endoscopic reports. The GI endoscopy procedures were categorized as diagnostic and therapeutic oesophagogastroduodenoscopy (OGD) and colonoscopy. The study participants were then subcategorized into those that had either only OGD or only colonoscopy or both. The endoscopic yield in relation to the type of bleed was also recorded. The diagnostic yield in this study was determined by relating the type of clinical bleeding to the significant endoscopic findings (18).

### Endoscopic Procedure

Before the procedure, each child was requested to have an overnight fast or a minimum fasting period of 6 hours before the procedure. The Karlz Storz video endoscope was used to perform the procedures, and each procedure was performed after venous access had been established for each child. During the procedure, the vital signs and oxygen saturation were monitored, and each procedure was performed under conscious sedation with intravenous (IV) midazolam ± ketamine and/or propofol with the help of the anesthetists. The endoscopic diagnosis was based on visual assessment documented by experienced endoscopists (1 pediatric and two adult gastroenterologists) and tissue biopsies were taken when necessary. Written informed consent was obtained for the procedure from the caregivers and assent was also obtained from the child where appropriate in children 7 years and above.

### Statistical Analysis

Data were analyzed with the Statistical Package for the Social Sciences (SPSS) version 25 (19). Continuous data were compared using the Mann-Whitney U test while the categorical data were compared with the chi-square test, respectively. Data were reported in numbers, percentages, and median (IQR). Receiver operating curves (ROC) were generated to test the ability of the Sheffield scores to predict rebleeds, mortality, and the need for endoscopic intervention. The area under the curve (AUC) was used to measure the discriminatory capability of the scores and was categorized as excellent = 0.9 to < 1, good = 0.8 to < 0.9, acceptable = 0.7 to < 0.8, and not good = 0.6 to <7 (20). The ROC is a plot of sensitivity against 1-specificity for a range of cutoff points of the Sheffield score to mortality, rebleeds, and endoscopic intervention. *P* < 0.05 was regarded as significant.

### Ethical Considerations

Ethical approval was obtained for the study from the Health Research and Ethics Committee of the Lagos University Teaching Hospital. The ethical approval assigned number is ADM/DCST/HREC/1888. Participant privacy and confidentiality of data management were ensured during and after the study.

## RESULTS

**FIGURE 1.**
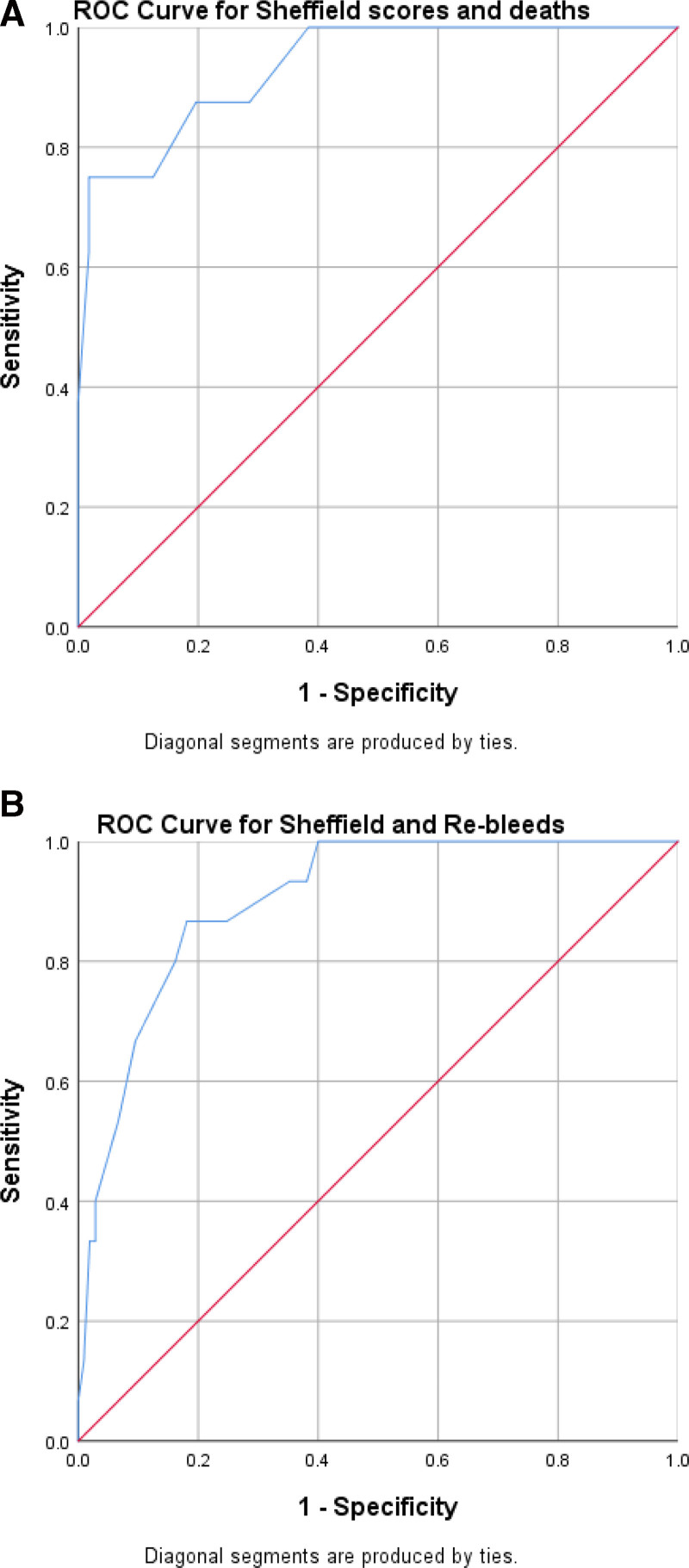
Flow chart showing the study participants.

One hundred and thirty children and adolescents with GI bleeding were referred for endoscopy during the study period; however, 10 were excluded due to incomplete clinical data. Thus, 120 subjects were enrolled in the study (Fig. 1).

### Baseline and Clinical Characteristics of Study Population

Table 1 highlights the baseline and clinical characteristics of the study participants. The majority of the participants were males (64, 53.3%) and belonged to the middle socioeconomic class.

**TABLE 1.** Clinical characteristics of the study participants according to gastrointestinal bleeding status

Parameter	Upper GI bleeding N = 91	Lower GI bleeding N = 29	Total N = 120	*P* value
Age (years) median (IQR)	7.00 (2.00-11.00)	9.00 (3.50-13.50)		0.062*
Age group (years)	N (%)	N (%)	N (%)	
<1	10 (10.9)	1 (3.4)	11 (9.2)	
1–<6	31 (34.1)	8 (27.6)	39 (32.5)	
6–12	32 (35.2)	12 (41.4)	44 (36.6)	0.475†
>12	18 (19.8)	8 (27.6)	26 (21.7)	
Gender				
Male	50 (54.9)	14 (48.3)	64 (53.3)	0.530†
Female	41 (45.1)	15 (51.7)	56 (46.7)	
Socioeconomic status				
High	15 (16.5)	7 (24.1)	22 (18.3)	
Middle	49 (54.9)	16 (55.2)	66 (55.0)	0.507*
Low	27 (28.6)	6 (20.7)	32 (26.7)	
Heart rate	107.00 (92.00-125.00)	87.00 (72.00-102.00)		**0.005** *
Duration of bleeding (days) (median [IQR])	2.00 (1.00-7.00)	5 (1.00-21.0)		
Medications used prior to presentation	N (%)	N (%)	N (%)	
None	64 (70.3)	22 (75.9)	86 (71.6)	0.812‡
NSAIDS	17 (18.7)	0 (0)	17 (14.2)	**0.015** ‡
Herbal preparations	5 (5.5)	0 (0)	5 (4.2)	0.334‡
Antibiotics	2 (2.2)	3 (10.3)	5 (4.2)	0.090‡
Others§	3 (3.3)	4 (13.8)	7 (5.8)	0.057‡
Syncope				
Yes	2 (2.2)	0 (0)	2 (6.9)	1.000‡
No	89 (97.8)	29 (100)	118 (93.1)	
Shock				
Present	8 (8.8)	1 (3.4)	9 (7.5)	0.685‡
Absent	83 (91.2)	28 (96.6)	111 (92.5)	
Abdominal pain				
Yes	29 (31.9)	15 (51.7)	44 (36.7)	0.075†
No	44 (48.3)	12 (41.4)	56 (46.7)	0.530†
Not known	18 (19.8)	2 (6.9)	20 (16.6)	0.152†
Diarrhea				
Present	1 (1.1)	13 (44.8)	14 (11.7)	**0.000**
Absent	90 (99.9)	16 (55.2)	106 (88.3)	
Blood transfusion				
Done	38 (41.8)	7 (24.1)	45 (37.5)	0.087†
Not done	53 (58.2)	22 (75.9)	75 (62.5)	
Resuscitation				
Done	13 (14.3)	2 (6.9)	15 (12.5)	0.294‡
Not done	78 (85.7)	27 (93.1)	105 (87.5)	
Outcome				
Died	6 (6.6)	2 (6.9)	8 (6.7)	0.977†
Survived	71 (78.0)	23 (82.8)	94 (80.0)	
Rebleeds/readmissions	14 (15.4)	4 (10.3)	18 (13.3)	

Bold values are statistically significant.

GI = gastrointestinal; IQR = Interquartile range.

* Mann-whitney U test.

† Chi-square test.

‡ Fischer’s exact test.

§One of the children with melena also had hematochezia; other medications: tegretol (1 patient), methotrexate and corticosteroid (1patient), and iron preparations (1patients).

### Study Participants with UGIB

Ninety-one (75.8%) of the participants presented with UGIB and the median [interquartile range (IQR)] age for these participants was 7.00 (2.00–11.00) years. Hematemesis only was seen in 63 (52.5%) of the total study participants, while hematemesis with melena was observed in 24 (20.0%). Melena alone was observed in 4 (3.3%) of the participants.

Forty-four (36.7%) study participants reported abdominal pain, and 29 of these presented with UGIB. Syncope was observed in 8.8% of the children with UGIB. Nine (7.9%) of the participants were brought in shock and the majority (8) of them had UGIB. The majority of the participants with rebleeds and deaths also presented with UGIB.

### Study Participants with LGIB

Twenty-nine (24.2%) presented with LGIB, that is, with hematochezia, and the median (IQR) of the participants was 9.00 (3.50–13.50) years. Fifteen (51.7%) participants with LGIB also reported recurrent abdominal pain while diarrhea was seen in 13 (44.8%) of the children. None of the children with LGIB presented with syncope and only 1 (2.2%) of them presented in shock.

### Other Clinical Findings in the Study Participants

Jaundice with hepatomegaly was observed in 6 (5.0 %) of the children; 5 of them had hemoglobinopathy (HbSS) while the last child had Biliary atresia. Splenomegaly with ascites was present in 3(2.5%) of the study participants. However, 4 (3.3%) children had weight loss and presented with LGIB.

### Comorbid Conditions Observed in the Study Participants

According to the clinical records, 71 of the study participants had underlying/comorbid conditions. (Supplemental Digital Content Table 1, http://links.lww.com/PG9/A136) A nasopharyngeal mass was observed in one of the children.

### Sheffield Scores in the Study Participants

The pattern of the Sheffield scores (a scoring system validated for UGIB) in the subjects is highlighted in Table 2. Sheffield score was ≥8 in 42 (46.1%) of the 91 children who presented with UGIB (hematemesis and melena). Using the adapted scoring system, 9 (31.0%) of the 29 children with LGIB (hematochezia) had scores ≥8.

**TABLE 2.** Sheffield Score of the participants and selected variables of clinical significance

Variable	Sheffield score ≥8 N = 51 N (%)	Sheffield score <8 N = 69 N (%)	Total N = 120 N (%)	*P* value
Type of Bleed				
UGIB				
Hematemesis	26 (50.9)	37 (53.6)	63 (52.5)	
Melena	0 (0.0)	4 (5.8)	4 (3.3)	0.016*
Hematemesis+melena	16 (31.4)	8 (11.6)	24 (20.0)	
LGIB				
Haematochezia†	9 (17.7)	20 (29.0)	29 (24.2)	
Age (years)				
<1	8 (15.7)	4 (5.8)	12 (10)	
1–<6	16 (31.4)	23 (33.3)	39 (32.5)	0.312‡
6–12	18 (35.3)	25 (36.2)	43 (35.8)	
>12	9 (17.6)	17 (24.6)	26 (21.7)	
Outcome				
Died	8 (15.7)	0 (0)	8 (6.7)	
Survived	27 (52.9)	67 (97.1)	94 (80.8)	0.000*
Rebleeds/readmissions	16 (31.4)	2 (2.9)	18 (12.5)	
Endoscopy done	N = 27	N = 43	N = 70	0.302
Retrospective (N)	24 (88.9)	40 (93.0)	64 (91.4)	
Prospective (N)	3 (11.1)	3 (7.0)	6 (8.6)	
Endoscopy not done	N = 24	N = 26	N = 50	
Retrospective (N)	21 (87.5)	26 (100)	47 (94.0)	
Prospective (N)	3 (12.5)	0 (0)	3 (6.0)	

LGIB = lower gastrointestinal bleeding; UGIB = upper gastrointestinal bleeding.

* Fischer’s exact test for *P* value.

† Chi-square statistics for *P* values.

‡ 2 of these children also had history of haematemesis.

All the participants who died had scores 8 and above. The Sheffield scores in relation to the endoscopic procedure also revealed that among the 51 participants with scores ≥8, 27 of the study participants underwent endoscopy (24 in the retrospective arm and 3 in the prospective arm) while endoscopy was not performed for 24 children (21 in the retrospective and 3 prospective arms respectively).

Of those children (69) with scores <8, 40 in the retrospective arm had endoscopy done while 26 did not have endoscopic evaluation.

### Endoscopic Procedures and Diagnosis in the Study Participants

Seventy (58.3%) of the participants underwent GI endoscopy and were most likely males. Fifty [50 (41.7%)] did not have GI endoscopy done. Major reasons for failure to have the procedure were lack of equipment, (21) financial constraints, (13) and religious inclinations (2). The children who did not have endoscopy were younger than those who had the procedure done. (Supplemental Digital Content Table 2, http://links.lww.com/PG9/A136).

Twenty-three (65.7%) of the 35 children who did not have the procedure due to lack of equipment had Sheffield scores ≥8, while 12 (38.3%) had scores <8. The scores of the 13 children who failed to have the procedure due to financial constraints were ≥8 in 6 (46.2%) and <8 in 7 (53.8%) children, respectively. The scores were <8 in those (2) who refused the procedure for religious reasons.

The endoscopic procedures were performed within 72 hours–7 days of presentation. Few therapeutic procedures were performed, and these included variceal banding (5), sclerotherapy (2), polypectomy (3), and hemoclip application (1) (Fig. 1).

### Endoscopic Diagnosis

The diagnostic findings in the 70 children who had endoscopy revealed that the prevalent findings in the children with UGIB were gastric erosions (15, 28.3%), esophageal varices (12, 22.6), and peptic ulcer disease (5, 9.4%) (Table 3). Five (29.4%) of the participants with LGIB had juvenile polyps and indeterminate colitis, respectively. Ulcerative colitis was seen in 4 (23.5%), while Crohn’s disease was present in one of the participants (5.9%) with LGIB.

**TABLE 3.** Endoscopic findings in the study participants who had gastrointestinal endoscopy

Endoscopic diagnosis	Frequency (%)
UGIB	53 (100)
Esophageal varices*	12 (22.6)
Gastritis†	11 (20.8)
Gastric erosions	15 (28.3)
Gastric ulcer	4 (7.5)
Gastric polyps	2 (3.8)
Duodenal ulcer	2 (3.8)
Nasopharyngeal mass	1 (1.9)
Hiatus hernia	1 (1.9)
Normal	5 (9.4)
LGIB	N (%) 17 (100)
Crohn’s disease	1 (5.9)
Ulcerative colitis	4 (23.5)
Indeterminate colitis	5 (29.4)
Juvenile polyps (rectal)	5 (29.4)
Hemorrhoids	2 (11.8)

LGIB = lower gastrointestinal bleeding; UGIB = upper gastrointestinal bleeding.

* One child also had gastric varices too.

† Two children also had duodenitis and 1 had esophagitis. +One child also had gastritis.

The overall endoscopic yield observed in this study was 76.9%. The yield obtained for hematochezia (88.2%) was higher than the yield for all the clinical types of GI bleeding (Supplemental Digital Content Table 3, http://links.lww.com/PG9/A136). Among the participants with UGIB, the yield was highest for the participants with hematemesis plus melena (83.3%). The only participant with melena had a significant finding on endoscopy; thus, the yield obtained was 100%.

### Performance of Sheffield Score in Predicting Clinical Outcome and Interventions in the Study Participants with UGIB

The performance of the significant Sheffield scores (≥8) in predicting clinical outcomes and intervention (variceal banding) in the participants with UGIB is demonstrated with ROC curves in Supplemental Digital Content Figure 1 and Figure 3, http://links.lww.com/PG9/A136 and with the AUC values in Supplemental Digital Content Table 4, http://links.lww.com/PG9/A136.

### Sensitivity and Specificity values for Sheffield Scores in the Study Participants with UGIB

The sensitivity and specificity values of Sheffield scores (≥8) for mortality were 100% and 69.8%, respectively (Supplemental Digital Content Table 4, http://links.lww.com/PG9/A136). For rebleeds, the sensitivity was high (91.7%) but the specificity was lower (73.8%). Variceal banding was the most common therapeutic endoscopy performed in this study, and the sensitivity value of the score for the procedure was the highest (100%) but the specificity was lower (67.8%).

The AUC values for the ROC curves of the Sheffield scores are also highlighted in Supplemental Digital Content Table 4, http://links.lww.com/PG9/A136. The AUC values showed good to excellent discriminatory ability of the scores to predict deaths, rebleeds and variceal banding.

## DISCUSSION

The findings in this study revealed that the majority of the subjects presented with UGIB (75.8%) rather than LGIB (24.2%). Hematemesis was only the most common clinical presentation of UGIB, while hematochezia was that of the lower GI bleeds. This is consistent with findings in several studies done in Europe (22–24), Asia, and some sub-Saharan African countries (21,25–27). In contrast to this finding Markus et al (3) in Germany documented hematochezia (LGIB) as the most common presentation. Thus, it appears that the clinical presentation of GI bleeding may vary with geographical location, and this is likely to be related to the prevalent disease in the area of study.

As documented in previous reports from Europe and Asia, GI bleeding was more prevalent in older children compared with infants with a predominant male sex predilection (21–29).

Abdominal pain was a prominent GI clinical feature in a third of the participants with UGIB in this study, while diarrhea was more prominent in those with LGIB. Some authors from Iran (29) and other developing countries (25–27) have reported a similar occurrence, and abdominal pain was attributed to *H. Pylori* infection. In this study, it is difficult to draw a similar conclusion because the *H. Pylori* status of all the children was not reported. Diarrhea, on the other hand, was significantly present in those with LGIB and this finding has also been reported previously by other authors (30,31). Other clinical findings in the study participants were related to their background illness. As noted by other authors, comorbid/underlying diseases are usually present in children with GI bleeding (21–28). The finding of a nasopharyngeal tumor in a child with hematemesis in this study suggests that nasopharyngeal lesions should be ruled out with this clinical presentation.

In terms of endoscopic evaluation, OGD and/or colonoscopy were performed on 70 (58.3%) children, in this study, indicating significant suboptimal use of GI endoscopy in this instance. According to the clinical criteria, endoscopy was indicated in all the study participants (120) recruited into the study because they all presented with GI bleeding. However, only 51 (42.5%) of them had significant Sheffield scores, indicating the need for endoscopic evaluation. Nevertheless, whether the endoscopy was performed was not related to Sheffield score or clinical outcomes.

The main reasons documented in this study for failure to perform endoscopy were lack of equipment and financial constraints. GI endoscopy is expensive, and the procedure is not subsidized or covered by health insurance in many low- and middle-income countries (LMICs) like Nigeria. Additionally, pediatric endoscopes are also not readily available in these settings. These findings reflect the barriers to this procedure in LMICs, where there is limited access to age-appropriate endoscopic facilities in addition to a dearth of skilled pediatric endoscopists (12,13). Thus, most procedures are usually diagnostic rather than therapeutic due to these various challenges. Thus, there is a need for continual advocacy to the relevant stakeholders for the provision of pediatric endoscopes and training and recruitment of personnel for pediatric endoscopy.

Nonvariceal bleeding (gastric erosions) was the most common endoscopic finding in the participants with UGIB, followed by esophageal varices, while peptic ulcer disease was seen in a smaller number of the participants. This is consistent with findings in some European studies (3,23) and Chinese studies (5). Huang et al (5) in 2003, in a retrospective review of the clinical records of 112 Chinese children over a 4-year period documented superficial mucosal gastric lesions in 44.6% of the children studied. However, a higher rate of peptic ulcer disease (gastric ulcers [9.8%], DUs [15.2%]) was observed in this study. The reasons for the latter finding may be related to the early presentation and endoscopic intervention in these Chinese children. However, this rarely occurs in the Nigerian setting, so the late presentation and healing of small ulcers before the procedure is done may obscure endoscopic diagnosis. Nonvariceal bleeding may also be due to the exposure to NSAIDs and herbal preparations which was observed in our setting, as reported previously by other authors (32).

In contrast, reports from Sudan (27), Iran (28) and India (33) documented esophageal varices as the most prevalent endoscopic finding in UGIB in these countries. This endoscopic finding was attributed to the high prevalence of schistosomiasis and possibly past umbilical catheterization for exchange blood transfusions in these settings. Some adult studies in southern and northern Nigeria have also reported similar observations (15,34). As documented by other authors from developing countries (21,30,35,36), juvenile polyps was a significant finding in the participants with LGIB in this study. However, the small number of participants with LGIB who had endoscopy makes it difficult to establish the most common etiology in this instance, thus larger studies are needed to evaluate the endoscopic findings of LGIB in Nigerian/African children. In Egypt, infectious enterocolitis was observed to be prevalent, followed by colorectal polyps (37).

The endoscopic yield obtained in this study for UGIB was 76.9 %, which is comparable to the yield (71.3%) documented in a recent study in Iran (38) in 136 children with UGIB. Other studies have reported higher yields (90%) in their series (24,28,29). This disparity in yields may be due to differences in the definition of the significant endoscopic findings. Additionally, as noted earlier, late presentation may also affect the yield of endoscopic diagnosis, especially for the mucosal lesions. The endoscopic yields obtained in this study support the recommendations for pediatric endoscopy in GI bleeding endorsed by various bodies (18,39,40).

Though the Sheffield scoring system was not developed for LGIB, children in this study were stratified using the system (14). The participants with significant cutoffs (≥8) had UGIB, indicating the necessity for early and urgent management for children with this symptom, especially those with acute massive bleeds who present in shock or who need blood transfusions.

Due to the lack of previous validation for LGIB and smaller numbers, we report the scores for our version of the Sheffield score used for the LGIB as exploratory data, and we did not examine ROC scores for these participants.

According to the Sheffield score, a significant proportion of the study participants who failed to have an endoscopy procedure due to lack of equipment and financial constraints would have benefited from the procedure because they had significant scores and appropriate diagnosis would also have been made. The ability of the scores to predict rebleeds, mortality, and the need for endoscopic interventions like variceal banding were applied mainly to the participants with UGIB and this revealed excellent and good values for the AUC (41) indicating that the scores were able to significantly discriminate for these parameters in children with GI bleeding and be useful in this setting. The sensitivity values obtained in this study were comparable to the values documented by Thompson et al (14) in Sheffield and Sari et al (42) in Indonesian children with UGIB, but the specificity values were lower than what has been documented in these 2 studies.

Though not the main aim of this study, we believe the use of an adaptation of the Sheffield system or a similar scoring system for lower GI bleeds may help in the triage of children who would need urgent endoscopic intervention or otherwise, and this needs to be further evaluated in a future study. The need for larger longitudinal studies to ascertain its usefulness in the African pediatric population is thus recommended.

## LIMITATIONS

This study was limited by the small number of participants and the inability to establish endoscopic diagnosis in all the children and adolescents who were referred for the procedure. In addition, the small number of participants with deaths and rebleeds may affect the reliability of the ROC curves.

## CONCLUSION

In conclusion, the clinical and endoscopic findings in pediatric GI bleeding in this resource-limited setting are similar to the findings in other regions of the world. The Sheffield scoring system may enable the stratification and identification of children with high-risk lesions and improve the management of pediatric GI bleeding in African settings where endoscopic facilities are not readily available.

## ACKNOWLEDGMENTS

We are grateful to the Nursing staff of the endoscopy unit of the Lagos University Hospital and to the anesthetists for their assistance in the completion of the endoscopic procedures.

## Supplementary Material


