# Supplementary material for: Gastrointestinal Bleeding in Children: The Role of Endoscopy and the Sheffield Scoring System in a Resource-Limited Setting
Source: JPGN Rep. 2023 Oct 5;4(4):e369. doi: 10.1097/PG9.0000000000000369 (PMC10684120; doi:10.1097/PG9.0000000000000369)
Supplement: Supplementary file 1 [file pg9-4-e369-s001.pdf]

APPENDIX 1

OGUNLESI MODIFICATION OF THE OYEDEJI SOCIAL CLASSIFICATION SCHEME

SOCIAL CLASS	PROFESSION	EDUCATIONAL ATTAINMENT
I	Professional, Senior public servants, Owners of large business concerns, Senior military officers, large scale contractors.	University graduates or equivalents
II	Non-academic professionals e.g. Nurses, Secondary school teachers, Secretaries, Owners of medium sized business. Intermediate grade public servants	School certificate holders and equivalent
III	Non manual skilled workers including clerks, typists, telephone operators. Junior school teachers. Drivers	Grade II teachers or equivalent
IV	Petty traders. Labourers. Messengers.	Primary certificate
V	Unemployed. Full time house wives. Students. Subsistence farmers.	No formal education

Ogunlesi TA, Dedeke IOF, Kuponiyi OT. Socio-economic classification of children attending specialist paediatric centres in Ogun state, Nigeria. Niger Med Pr. 2008; 54(1):21–25.

APPENDIX 2

Sheffield Scoring System

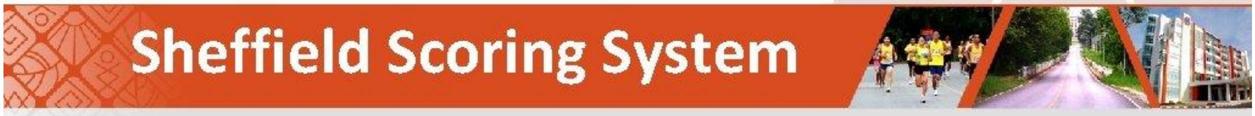

TABLE 6. Idealised scoring system

History taking
Significant preexisting condition: 1
Presence of melaena: 1
History of large amount of haematemesis: 1
Clinical assessment
HR >20 from the mean HR for age: 1
Prolonged capillary refill: 4
Laboratory findings
Hb drop of >20 g/L: 3
Management and resuscitation
Need for a fluid bolus: 3
Need for blood transfusion (Hb of <80 g/L): 6
Need for other blood product: 4
Total score 24: cutoff 8
Interventional group: true-positive = 31, false-negative = 4
Noninterventional group: true-negative = 31, false-positive = 3
Sensitivity: 88.57%, 95% CI 73.24–96.73
Specificity: 91.18%, 95% CI 76.30–98.04
PPV: 91.18%, 95% CI 76.30–98.04
NPV: 88.57%, 95% CI 73.24–96.73

CI = confidence interval; Hb = haemoglobin; HR = heart rate; NPV = negative predictive value; PPV = positive predictive value.

Thomson MA, et al. JPGN 2015;60: 632–36.

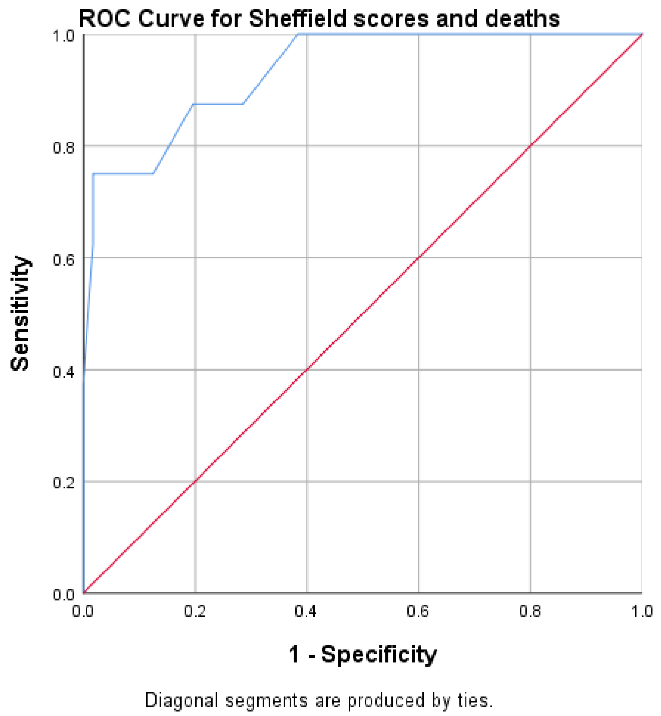

Figure 1a: ROC curve for Sheffield scores and deaths in the study participants

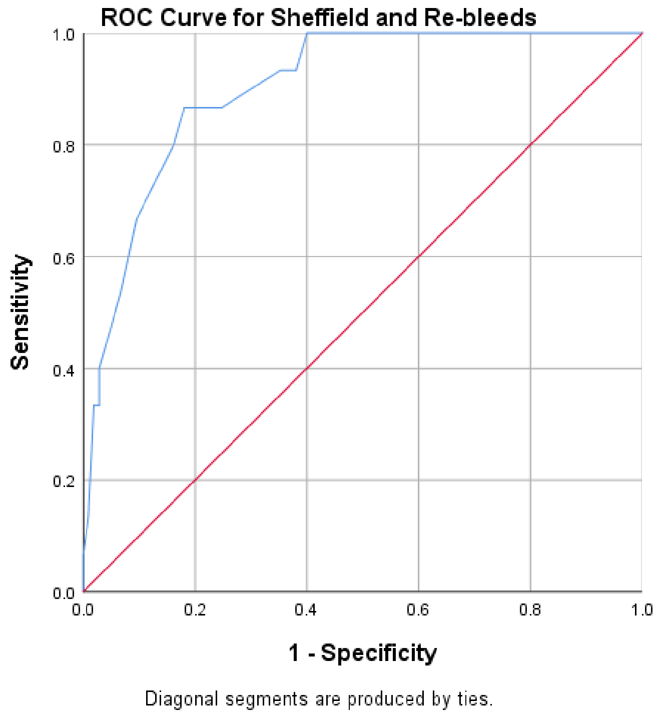

Figure 1b: ROC curve for Sheffield scores and re-bleeds in the study participants

Table 1: Co-morbid conditions observed in the study participants

Underlying/Comorbid Condition	Frequency
Biliary Atresia	1
Chronic Kidney disease secondary to Nephrocalcinosis, Hydronephrosis	2
Congenital heart disease (ASD + Pulmonary stenosis)	1
Covid19 and MIS	2
Cholelithiasis	1
Food Allergy	2
GERD	1
Juvenile Idiopathic Arthritis	1
Hepatitis B	1
Hodgkin's lymphoma	1
Malaria	6
Metastatic Retinoblastoma	1
Portal Hypertension	5
Nasopharyngeal Mass	1
Sickle cell anaemia	5
Retroviral disease	2
Portal vein thrombosis	4
Sepsis	33
None	49

ASD- Atrial septal defect, MIS-Multisystem inflammatory response, GERD-Gastroesophageal reflux disease.

Table 2: Characteristics of study participants in relation to GI endoscopy status.

UPPER GI BLEEDING				
**Parameter**	**Endoscopy yes n=53 N (%)**	**Endoscopy no N=38 N (%)**	**Total N=91 N (%)**	**P value**
**Age group(years)**				
<1	4(7.6)	7(18.4)	11(12.1)	
1-<6	13(24.5)	18(47.4)	31(34.1)	**0.015**
6-12	22(41.5)	9(23.7)	31(34.1)	
>12	14(26.4)	4(10.5)	18(19.7)	
**Gender**				
Male	21(39.6)	29(76.3)	50(54.9)	**0.000**
Female	32(60.4)	9(23.7)	41(45.1)	
**Died**				
Yes	3(5.7)	3(7.9)	6(6.6)	0.671
No	50(94.3)	35(92.1)	85(93.4)	
**Rebleeds/Readmissions**				
Yes	8(15.1)	6(15.8)	14(15.4)	0.927*
No	45(84.9)	32(84.2)	77(84.6)	
**Sheffield score**				
>8	24(45.3)	19(50.0)	43(47.3)	0.656
<8	29(54.7)	19(50.0)	48(52.7)	
**Socioeconomic status**				
High	7(13.2)	8(20.5)	15(16.5)	0.571
Middle	31(58.5)	19(51.3)	50(54.9)	
Low	15()	11(28.2)	26(28.6)	
**LOWER GI BLEEDING**				
**Parameter**	**Endoscopy (yes) n=17 N (%)**	**Endoscopy(No) n=12 N (%)**	**Total n=29 N (%)**	**P value**
**Age**				
<1	0 (0.0)	1(9.1)	1(3.4)	0.146*
1-<6	4 (22.2)	4(36.4)	8(27.6)	
6-12	6 (33.3)	6(54.5)	12(41.4)	
>12	7 (44.4)	1(0.0)	8(27.6)	
**Gender**				
Male	10(58.8)	4(33.3)	14(48.3)	0.176
Female	7(41.2)	8(66.7)	15(51.7)	
**Died**				
Yes	1(5.9)	1(8.3)	2(6.9)	0.291*
No	16(94.1)	11(91.7)	27(93.1)	
**Rebleeds/Readmissions**				
Yes	3(17.6)	1(8.3)	4(13.8)	0.473*
No	14(82.4)	11(91.7)	25(86.2)	
**Sheffield score**				
>8	3(17.6)	5(41.7)	8(27.6)	0.154
<8	14(82.4)	7(58.3)	21(72.4)	
**Socioeconomic status**				
High	6(35.3)	1(8.3)	7(24.1)	0.154*
Middle	9(52.9)	7(58.3)	16(55.2)	
Low	2(11.8)	4(33.3)	6(20.7)	

Chisquare stastic for pvalue,*Fischer's exact test for p valu

Table 3: Type of Bleed and Endoscopic yield in the study participants

**Type of Bleed**	**Number of children with bleed**	**Number of participants with significant endoscopic finding**	**Endoscopic yield %**
**All Haematemesis	52	40	76.9
Haematemesis only	41	36	87.8
Haematemesis and melena	12	10	83.3
Melena only	1	1	100.0
Haematochezia only	17	15	88.2

**All Haematemesis=Haematemesis only+Haematemesis with melena+Haematemesis with haematochezia from UGIB

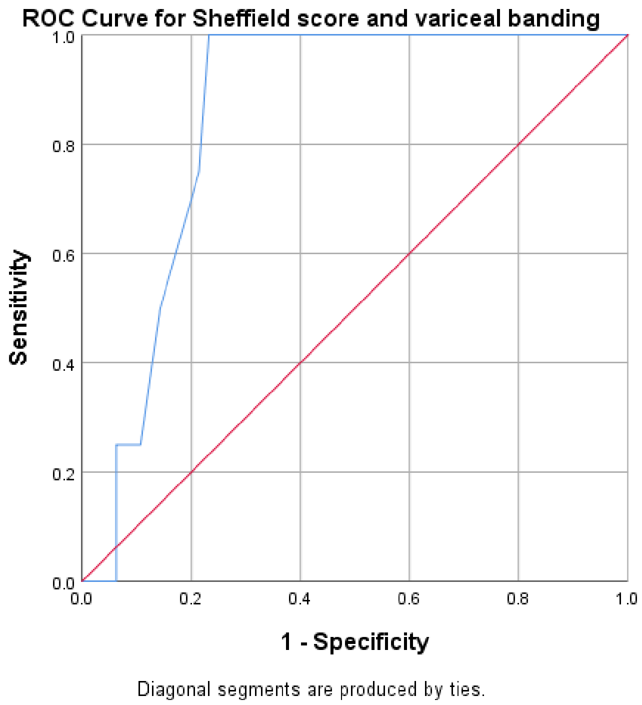

Figure 2: Receiver operating curve for Sheffield score and variceal banding in the participants with UGIB.
